# Precipitation Changes Regulate Plant and Soil Microbial Biomass *Via* Plasticity in Plant Biomass Allocation in Grasslands: A Meta-Analysis

**DOI:** 10.3389/fpls.2021.614968

**Published:** 2021-02-25

**Authors:** Chunhui Zhang, Nianxun Xi

**Affiliations:** ^1^State Key Laboratory of Plateau Ecology and Agriculture, Qinghai University, Xining, China; ^2^Department of Ecology, Sun Yat-sen University, Guangzhou, China

**Keywords:** aboveground-belowground interaction, asynchrony, carbon stock, grasslands, optimal biomass allocation, precipitation

## Abstract

In theory, changes in the amount of rainfall can change plant biomass allocation and subsequently influence coupled plant-soil microbial processes. However, testing patterns of combined responses of plants and soils remains a knowledge gap for terrestrial ecosystems. We carried out a comprehensive review of the available literature and conducted a meta-analysis to explore combined plant and soil microbial responses in grasslands exposed to experimental precipitation changes. We measured the effects of experimental precipitation changes on plant biomass, biomass allocation, and soil microbial biomass and tested for trade-offs between plant and soil responses to altered precipitation. We found that aboveground and belowground plant biomass responded asynchronically to precipitation changes, thereby leading to shifts in plant biomass allocation. Belowground plant biomass did not change under precipitation changes, but aboveground plant biomass decreased in precipitation reduction and increased in precipitation addition. There was a trade-off between responses of aboveground plant biomass and belowground plant biomass to precipitation reduction, but correlation wasn't found for precipitation addition. Microbial biomass carbon (C) did not change under the treatments of precipitation reduction. Increased root allocation may buffer drought stress for soil microbes through root exudations and neutralize microbial responses to precipitation reduction. However, precipitation addition increased microbial biomass C, potentially reflecting the removal of water limitation for soil microbial growth. We found that there were positive correlations between responses of aboveground plant biomass and microbial biomass C to precipitation addition, indicating that increased shoot growth probably promoted microbial responses via litter inputs. In sum, our study suggested that aboveground, belowground plant biomass and soil microbial biomass can respond asynchronically to precipitation changes, and emphasizes that testing the plant-soil system as a whole is necessary for forecasting the effects of precipitation changes on grassland systems.

## Background

Grasslands represent the largest terrestrial biome, playing crucial roles in global carbon (C) cycling because they have higher soil C contents than other vegetation types for a given climate regime (Anderson, 1991). Plant productivity and microbial biomass C are two key factors in determining grassland C cycling. However, grassland C cycling may be largely variable, because of an increased frequency of wetter or drier years in future (Knapp and Smith, 2001). To date, the coupling responses of plants and soil microbes to precipitation changes remain a knowledge gap. There is an urgent need to fill this gap to acquire a comprehensive understanding of grassland ecosystems in the context of climate change.

**Figure 1:**
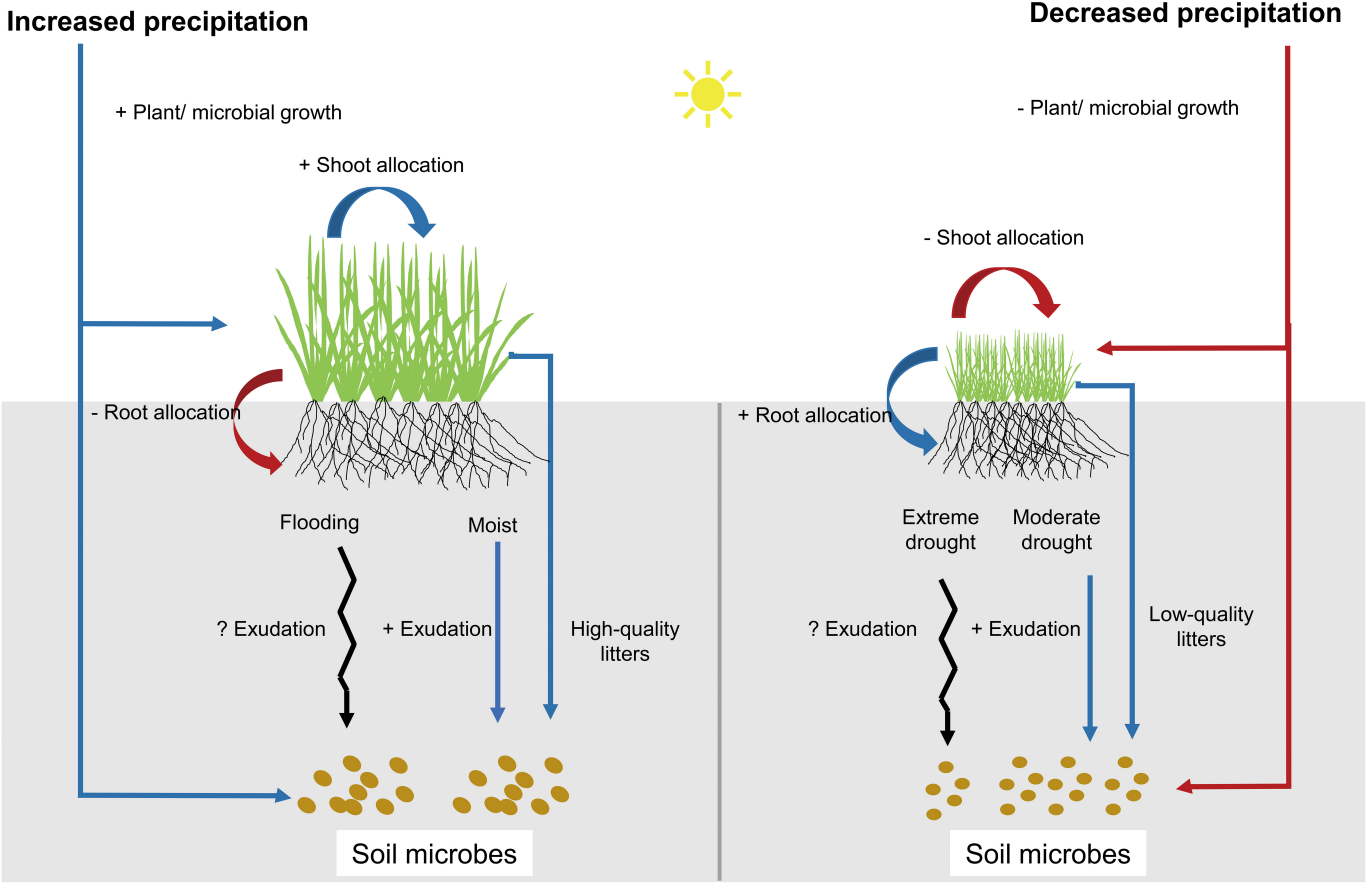
Graphical depiction of how precipitation changes influence aboveground and belowground biomass *via* plasticity in plant biomass allocations. “+” and “–” indicate “increasing” and “decreasing” respectively. Black arrows indicate that there are no consistent conclusions on these ecological processes.

Previous studies have illustrated that aboveground (APB) and belowground plant biomass (BPB) responded differently to precipitation changes, regulated by shifts in plant biomass allocation patterns (Byrne et al., 2013; Wilcox et al., 2017). Optimal allocation theory asserts that plants should allocate biomass to the organ that acquires the most limiting resource (Bloom et al., 1985; Gleeson and Tilman, 1992; Giardina et al., 2003). Under decreased precipitation (DPPT) conditions, plants may increase the allocation of carbohydrates to roots to maximize soil resource uptake, thus minimizing BPB loss while exacerbating APB loss (Figure 1). Under increased precipitation (IPPT) conditions, plants may increase aboveground growth to maximize light capture, resulting in greater aboveground responses than belowground (Figure 1). In principle, there are trade-offs between APB and BPB in the responses to either DPPT or IPPT, because in plants allocation of photosynthetic production is a zero-sum dynamic, i.e., increased allocation of production to shoots or roots must be at the expense of other organs (Mccarthy and Enquist, 2007).

Despite an increasing number of studies testing plant above- and belowground responses to altered precipitation, one critical knowledge gap is the combined response of plants and soil microbes to changes in precipitation amount. This lacuna could lead to a biased understanding of grassland function and service under climate change because soil microorganisms play a key role in carbon-cycling processes, such as litter decomposition and greenhouse gas emissions (van der Heijden et al., 2008; Bardgett and van der Putten, 2014). There are many reports showing that rainfall amounts can strongly influence soil microbial growth and community structure by changing soil moisture (Williams, 2007; Hueso et al., 2012; Ochoa-Hueso et al., 2018). Changes in the amount of rainfall also indirectly influence microbial communities by regulating litter inputs and changing root exudations (Figure 1) (Nielsen and Ball, 2015; Luo et al., 2017; Williams and de Vries, 2020). For instance, under moderate drought stress, plants may increase allocations of assimilated C into root soluble sugars that can be released as root exudates, thereby influencing soil microbial C uptakes (Preece and Peñuelas, 2016; Karlowsky et al., 2018a). However, root exudations and its consequence on soil microbes can be highly variable during extreme drought stress (Preece and Peñuelas, 2016). Moderate increases in soil moisture may also enhance root exudations, as plants increase root growth and release root exudates that may have enzymatic properties and can enhance degradation of organic matters and microbial activity (Dijkstra and Cheng, 2007; Canarini et al., 2019). However, high soil moisture/flooding may lead to hypoxia and shift root respiration from aerobic to anaerobia, thereby complicating the patterns of root exudations (Badri and Vivanco, 2009). Although there are strong associations between plants and soil microbes via exchanges at root-soil interfaces, plants and microbes may respond asynchronously to soil resource availability due to contrasting life history strategies (Xi et al., 2014). Soil microorganisms can adapt to changes in soil moisture more rapidly than plants due to their fast growth, considerable capacity for osmotic adjustment under fluctuating soil moisture, speedy community composition shifts, and potential for contemporary evolution (Schimel et al., 2007; Lau and Lennon, 2011). The asynchrony between plant and microbial biomass may have significant implications for the competitive balance of plants and soil microbes, as well as for the regulation of biogeochemical cycling (Karlowsky et al., 2018a; Williams and de Vries, 2020).

Recently, a growing number of field grassland experiments have recorded APB, BPB, or MBC responses to precipitation amounts in individual ecosystems, providing a valuable opportunity to test shifts in plant biomass allocation and to compare plant versus soil microbial responses to altered precipitation at grasslands. To achieve a comprehensive understanding of the responses of plant-soil systems to precipitation changes in grassland ecosystems, we synthesized results from 499 experimental observations using a meta-analytical method. We searched for studies that measured plant biomass and microbial biomass carbon (MBC) under different manipulated precipitation amounts. Data were extracted and analyzed to address the following hypotheses: (1) microbial biomass C has greater responses to precipitation increases and decreases than plant biomass; (2) there is a trade-off between plant above- and belowground responses to precipitation increases and decreases; (3) responses of microbial biomass C and plant biomass are positively correlated.

## Methods

### Data Collection

We searched the literature using Web of Science (http://isiknowledge.com) on 1st August 2019. Two sets of search terms were used to obtain papers related to primary productivity and soil microbial biomass in response to experimental precipitation manipulations in grassland ecosystems that were published between 1st January 1900 and 1st August 2019. The first set of terms was “(‘plant growth” OR “primary product^*^’ OR ‘plant product^*^’ OR ‘ANPP’ OR ‘BNPP’) AND (‘altered precipitation’ OR ‘drought’ OR ‘decreased precipitation’ OR ‘increased precipitation’ OR ‘increased summer precipitation’ OR ‘decreased summer precipitation’ OR ‘water addition’ OR ‘water reduction’ OR ‘water treatment^*^’) AND (‘herbaceous’ OR ‘grass^*^’) AND (‘experiment^*^’ OR ‘treatment^*^’).” The second set of terms is “(‘microbial biomass’) AND (‘altered precipitation’ OR ‘drought’ OR ‘decreased precipitation’ OR ‘increased precipitation’ OR ‘increased summer precipitation’ OR ‘decreased summer precipitation’ OR ‘water addition’ OR ‘water reduction’ OR ‘water treatment^*^’) AND (‘herbaceous’ OR ‘grass^*^’) AND (‘experiment^*^’ OR ‘treatment^*^’).” We selected papers based on the following criteria: (a) experiment was conducted in the field; (b) the magnitude of precipitation changes was clearly described; (c) studies recorded paired responses to precipitation changes (i.e., APB vs. BPB, or APB vs. MBC, or BPB vs. MBC); (d) no other forcing factors (e.g., nutrient addition, warming) were applied in the precipitation treatments.

Our dataset consisted of experimental studies that are set up across 32 sites which are located in Asia, North America, Africa and Oceania (Supplementary Figure 1). For plant biomass, we focused on APB and BPB, and this dataset included 65 published papers (Supplementary Figure 2 and Supplementary Appendix 1). MBC is a commonly used indicator for microbe biomass and is widely measured in studies of grassland ecology (Li et al., 2004; Treseder, 2008; He et al., 2020). Therefore, for soil microbial biomass, we focused on MBC, and this dataset included 30 published papers (Supplementary Figure 2 and Supplementary Appendix 2). Ultimately, there were 33 case studies in which MBC and APB were paired [6 DPPT case studies (11 experimental observations) + 27 IPPT case studies (35 experimental observations)]. There were 19 case studies in which MBC and BPB were paired [(4 DPPT case studies (4 experimental observations) + 15 IPPT case studies (15 experimental observations)], and 60 case studies where APB and BPB were paired [23 DPPT case studies (34 experimental observations) + 37 IPPT case studies (54 experimental observations)].

### Effect Sizes

Effect size was calculated using a natural log-transformed response ratio (RR) for each observation (Hedges et al., 1999):

$$\begin{array}{l} RR=\text{Ln}\left(\frac{\bar{X_{t}}}{\bar{X_{c}}}\right) \end{array}$$

where $\bar{X_{t}}$ and $\bar{X_{C}}$ are the means of biomass (APB, BPB, or MBC) or soil moisture in changed rainfall (IPPT or DPPT) and ambient treatments, respectively. Its variance (*v*_*RR*_) was calculated as (Hedges et al., 1999):

$$\begin{array}{l} v_{RR}=\frac{s_{t}^{2}}{n_{t}X_{t}^{2}}+\frac{s_{c}^{2}}{n_{c}X_{c}^{2}} \end{array}$$

where *n*_*t*_ and *n*_*c*_ are the sample size of the concerned variable in the treatment and control, respectively; *s*_*t*_ and *s*_*c*_ are the standard deviations of the concerned variable in the treatment and control groups, respectively.

We also calculated effect size of aboveground: belowground biomass ratio in responses to DPPT or IPPT (*D*):

$$\begin{array}{l} D=\ln\left(\frac{\bar{APB_{t}}}{\bar{BPB_{t}}}\right)-\ln\left(\frac{\bar{APB_{c}}}{\bar{BPB_{c}}}\right)=\ln\left[\frac{\left(\frac{\bar{APB_{t}}}{\bar{BPB_{t}}}\right)}{\left(\frac{\bar{APB_{c}}}{\bar{BPB_{c}}}\right)}\right]\\ \;=\ln\left[\frac{\left(\frac{\bar{APB_{t}}}{\bar{APB_{c}}}\right)}{\left(\frac{\bar{BPB_{t}}}{\bar{BPB_{c}}}\right)}\right] \end{array}$$

$$\begin{array}{l} =\ln\left(\frac{\bar{APB_{t}}}{\bar{APB_{c}}}\right)-\ln\left(\frac{\bar{BPB_{t}}}{\bar{BPB_{c}}}\right)=RR_{APB}-RR_{BPB} \end{array}$$

where $\bar{APB_{t}}$ and $\bar{BPB_{t}}$ are the means of above- and belowground biomass in changed rainfall treatments (IPPT or DPPT). $\bar{APB_{C}}$ and $\bar{BPB_{C}}$ are the means of above- and belowground biomass in ambient rainfall treatments. *RR*_*APB*_ and *RR*_*BPB*_ are effect sizes for aboveground and belowground biomass respectively. Consequently, the positive/negative sign of *D* indicates that altered precipitation increases or reduces biomass allocation to aboveground components. Its variance (*v*_D_) was calculated as (Borenstein, 2009):

$$\begin{array}{l} v_{D}=\frac{v_{RR\left(APB\right)}}{n_{RR\left(APB\right)}}+\frac{v_{RR\left(BPB\right)}}{n_{RR\left(BPB\right)}} \end{array}$$

where *n*_*RR*(*APB*)_ and *n*_*RR*(*BPB*)_are sample sizes of *RR*_*APB*_ and *RR*_*BPB*_ in altered precipitation and ambient treatments, respectively; *v*_*RR*(*APB*)_ and *v*_*RR*(*BPB*)_ are the variances of *RR*_*APB*_ and *RR*_*BPB*_ respectively.

### Statistical Analyses

We calculated the average response ratio of APB, BPB, MBC, soil moisture, and plant aboveground: belowground biomass ratio using meta-analytic mixed models with case study as a random factor. We performed pairwise comparisons between effect sizes of APB, BPB, and MBC using meta-analytic mixed models with case study as a random factor. We set up linear mixed-effects models, with case study as a random factor, to test correlations between effect sizes of MBC, APB, and BPB. Because of the low number of observations (<5), we did not test the relationships between effect sizes of MBC and BPB to DPPT. The case study was designated as a random effect within the mixed-effects model to account for pseudo-replication originating from studies spanning multiple years.

We tested publication bias of the sensitivity based on the rank correlation test for funnel plot asymmetry, and did not detect publication bias (Supplementary Table 1). All analyses were conducted in R (R Core Team, 2019), and meta-analytic mixed-effects models and linear mixed-effects models were conducted using the *metafor* (Viechtbauer, 2010) and *lme4* (Bates et al., 2015) package, respectively.

## Results

### Effects of Decreased Precipitation on Plant Biomass, Microbial Biomass C, and Soil Moisture

**Figure 2:**
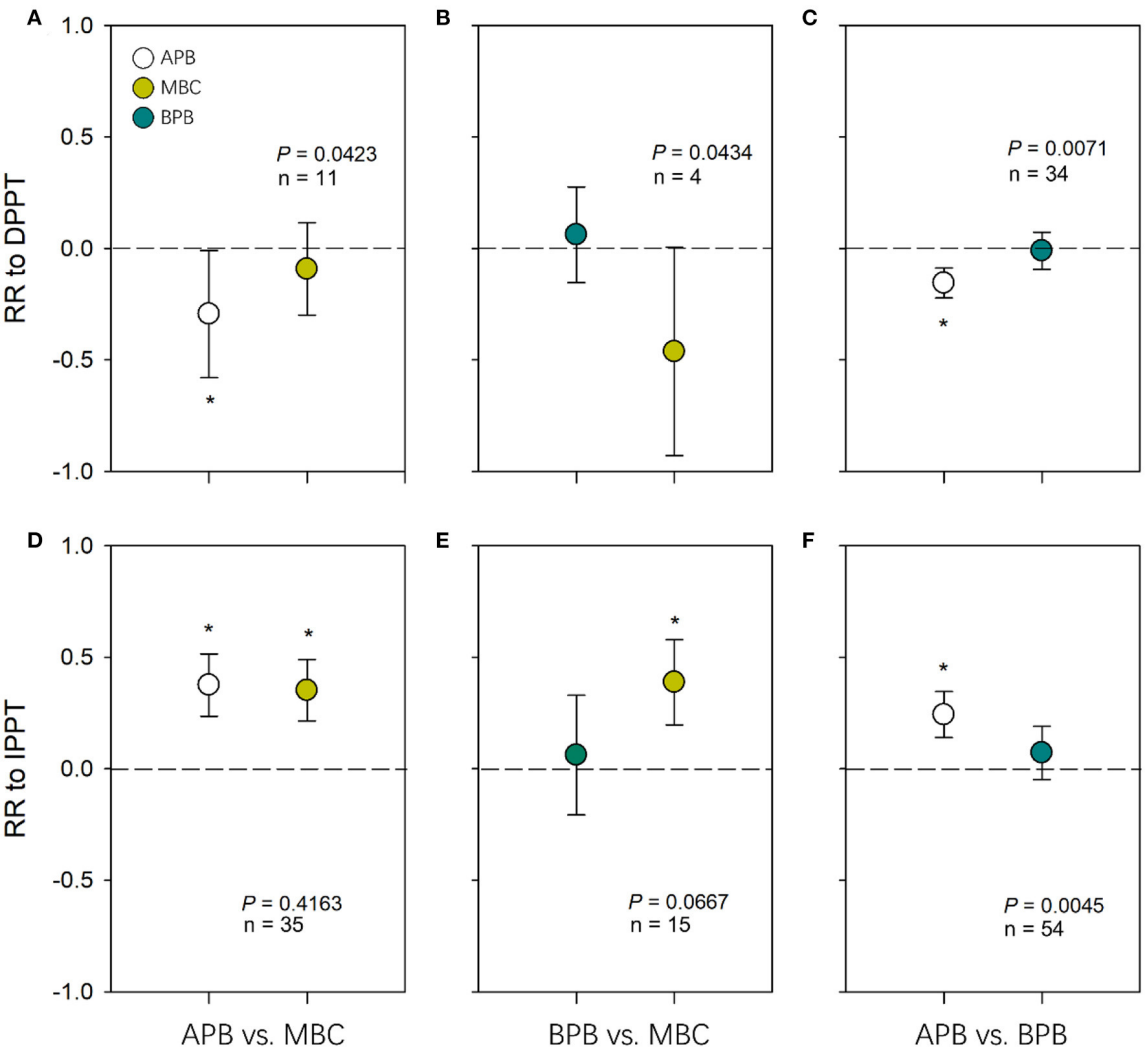
Pairwise comparisons of response ratio between APB, BPB, and MBC in response to DPPT **(A, B, C)** and IPPT **(D, E, F)**. The error bars represent 95% confidence intervals. *P* values are given to show the significance of pairwise comparisons. Asterisks indicate the average effect sizes as they differ from zero (*P* < 0.05). The dotted lines indicate the effect size of zero. DPPT, decreased precipitation; IPPT, increased precipitation.

Effect size of APB to DPPT was significantly negative (*P* < 0.05), and effect sizes of MBC and BPB to DPPT were not different from 0 (*P* > 0.05) (Figures 2A–C). There were significant differences between effect sizes of MBC and APB in responses to DPPT (*Q*_*M*_ = 4.12, *df* = 1, *P* = 0.0423); effect size of APB to DPPT was negative compared to that of MBC (Figure 2A). There were differences between effect sizes of MBC and BPB in responses to DPPT (*Q*_*M*_ = 4.08, *df* = 1, *P* = 0.0434); effect size of MBC to DPPT tended to be negative compared to that of BPB (Figure 2B). Effect size of APB to DPPT was negative compared to that of BPB (*Q*_*M*_ = 7.24, *df* = 1, *P* = 0.0071; Figure 2C). Effect size of soil moisture to DPPT was significantly negative (*P* < 0.05; Supplementary Figure 3).

### Effects of Increased Precipitation on Plant Biomass, Microbial Biomass C, and Soil Moisture

Effect sizes of MBC and APB to IPPT were significantly positive (*P* < 0.05), and effect sizes of BPB to IPPT were not different from 0 (*P* > 0.05) (Figures 2D–F). The effect size of APB to IPPT was positive compared to that of BPB (*Q*_*M*_ = 8.08, *df* = 1, *P* = 0.0045; Figure 2F). There were no differences in effect sizes to IPPT between APB and MBC (*Q*_*M*_ = 0.66, *df* = 1, *P* = 0.4163; Figure 2D) or between BPB and MBC (*Q*_*M*_ = 3.36, *df* = 1, *P* = 0.0667; Figure 2E). Effect size of soil moisture to IPPT was significantly positive (*P* < 0.05; Supplementary Figure 3).

### Shifts in Plant Aboveground: Belowground Biomass Ratio During Altered Precipitation

**Figure 3:**
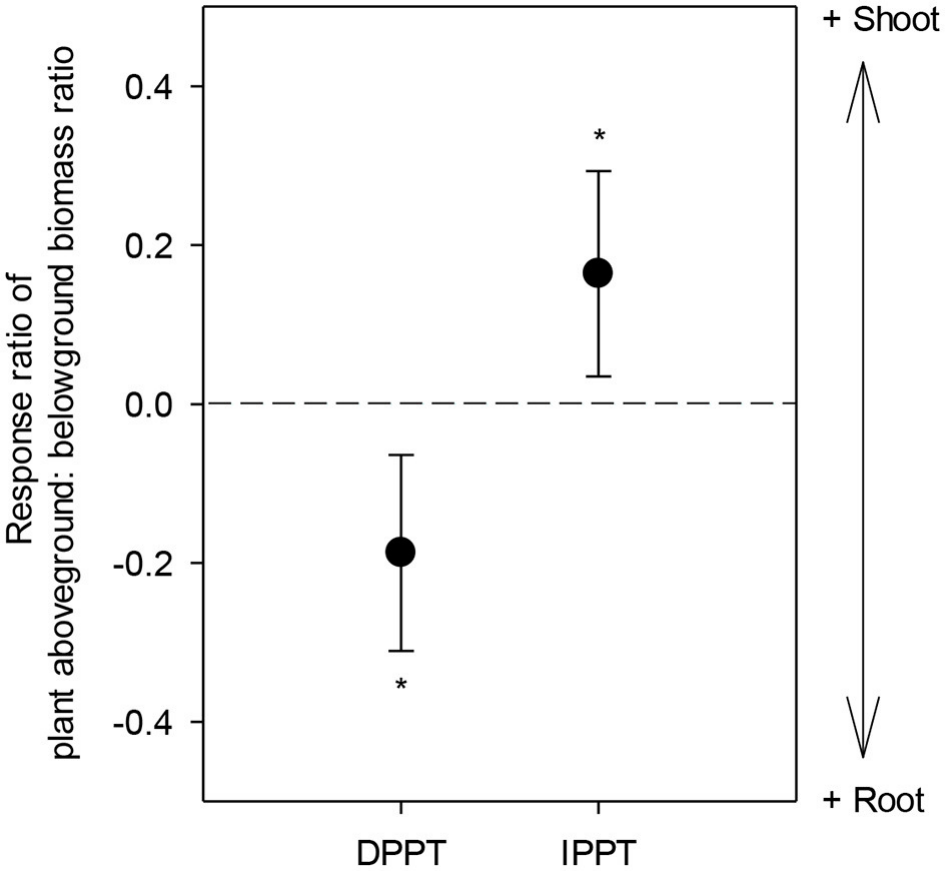
Response ratio of plant aboveground: belowground biomass ratio (i.e., D) in response to DPPT and IPPT. The error bars represent 95% confidence intervals. Asterisks indicate average response ratios as they differ from zero (*P* < 0.05). The dotted line indicates the effect size of zero. DPPT, decreased precipitation; IPPT, increased precipitation.

Effect size of plant aboveground: belowground biomass ratio (i.e., D) to DPPT was significantly negative (*P* = 0.0029), while effect size of its responses to IPPT was positive (*P* = 0.0128) (Figure 3).

### Correlations Between MBC, APB, and BPB

**Figure 4:**
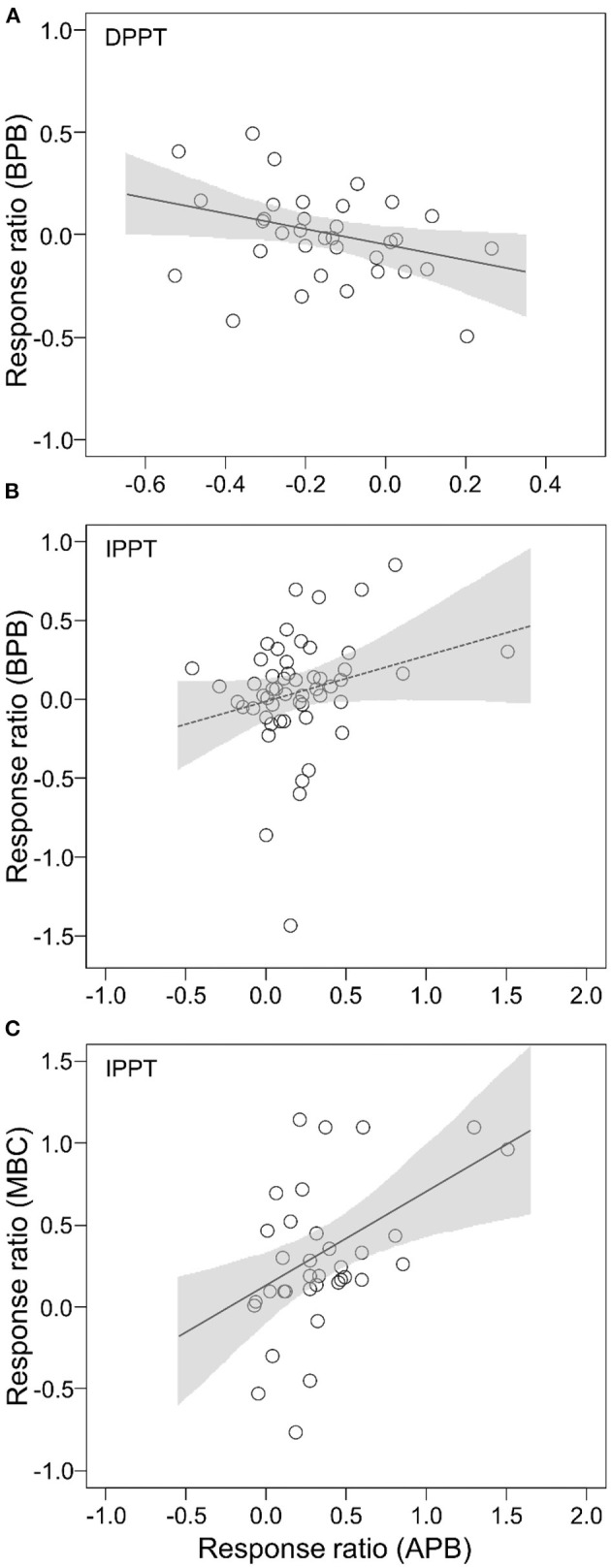
Relationships between effect sizes of BPB, APB, and MBC to altered precipitation: **(A)** effect sizes of APB vs. BPB to DPPT; **(B)** effect sizes of APB vs. BPB to IPPT; **(C)** effect sizes of APB vs. MBC to IPPT. The shaded areas represent 95% confident intervals.

There was a negative relationship between effect size of APB and BPB to DPPT (slope = −0.38, *P* = 0.0384; Figure 4A; Supplementary Table 2). There was a marginally significant correlation between effect size of APB and BPB to IPPT (slope = 0.30, *P* = 0.0715; Figure 4B; Supplementary Table 2). The effect size of MBC to IPPT was positively correlated with that of APB (slope = 0.58, *P* = 0.0023; Figure 4C; Supplementary Table 2). We did not detect significant correlation between the effect sizes of APB and MBC to DPPT (*P* = 0.2983), or between the effect sizes of BPB and MBC to IPPT (*P* = 0.2963).

### Correlations Between Soil Moisture and Precipitation Changes

Precipitation changes were significantly positively related to soil moistures (slope = 0.69, *P* < 0.0001; Supplementary Figure 4).

## Discussion

Plants and soil biota are inter-linked in mediating soil C cycling in grasslands (Bardgett et al., 2008; Ficken and Warren, 2019), but how plant-soil systems respond to altered precipitations has not been clarified. We filled this knowledge gap using a meta-analysis of published experimental studies. This meta-analysis has produced the key finding that plant biomass allocation determines plant-soil systems to precipitation changes, and this can increase our understanding of the likely influences of future climate change on grassland ecosystems.

### Effects of Precipitation Reduction on Plants and Soil Microbes

Our results indicated that APB was more sensitive than BPB in response to decreased precipitation (Figure 2A). We provided the first evidence that experimental drought manipulations caused greater allocation to roots, while added precipitations increased biomass allocation to shoots (Figure 3). This finding was consistent with theoretical prediction (Gleeson and Tilman, 1992) and empirical evidence for global patterns of biomass allocation across environmental gradients (Mccarthy and Enquist, 2007; Poorter et al., 2012). The plasticity in plant biomass allocation probably caused different responses of APB vs. BPB in responses to altered precipitation. Increased root allocation can promote soil water and nutrient capture and, therefore, buffer drought effects on BPB. Therefore, increasing APB responses unavoidably decreased BPB responses due to shifts in biomass allocations between above versus belowground production.

Contrary to what we predicted, we found that MBC tended to be more sensitive to DPPT than BPB but less sensitive than APB (Figure 2A). In principle, MBC responses to precipitation changes are directly driven by microbial eco-physiological characters or indirectly by soil conditions (such as available water, substrate, or nutrients for microbes) changes (Schimel et al., 2007). Under DPPT, plants could increase production of soluble root sugars for supporting the survival of roots through providing C for respiration, or for enhancing fine root growth to increase plant access to deep soil water (Karlowsky et al., 2018a,b; Hasibeder et al., 2015). Soluble root sugars may be linked with root exudations (Karlowsky et al., 2018a), thereby increasing the substrate available for soil microbes and buffer environmental stress for the microbial communities (Bloor et al., 2018). We also cannot rule out the possibility that changes in microbial community composition increase the resistance of whole soil communities to drought, because previous studies have suggested that drought-tolerant taxa, such as fungi or microbes with *K* strategy, increase their relative abundances in drought conditions (Manzoni et al., 2012; de Vries and Shade, 2013; Ren et al., 2018).

### Effects of Precipitation Addition on Plants and Soil Microbes

Under IPPT, overall effects of increased precipitation resulted in increased shoot allocation (Figures 2B, 3), reflecting light may be the more limiting factor for plants than soil water. Under wet conditions, the longevity of live roots is typically greater (Facette et al., 1999), reducing the need/ space for increased BPB to renew root systems (Hayes and Seastedt, 1987). Other studies showed that, under wetter conditions, saturated soil moisture conditions might limit root development (Kozlowski, 1997). We did not detect significant correlation between response ratio of APB and BPB to IPPT (Figure 4B), mirroring that experimental precipitation addition did not completely remove water limitation for plants. However, we suggest that plasticity in biomass allocation was likely to be the important factor driving different APB vs. BPB responses to IPPT and DPPT, and BPB responses to IPPT may be associated with root physiological changes.

In contrast with DPPT, IPPT stimulated microbial biomass (Figure 2B), reflecting the asymmetry of MBC responses to IPPT and DPPT. IPPT could directly increase soluble substrate availability for microbial communities through enhancing soil moisture (Schimel et al., 2007; Borken and Matzner, 2009). We did not detect a significant difference in response ratios of MBC vs. APB or BPB to IPPT (Figure 2B), and this finding may simply reflect the fact that increased soil moisture linearly mitigated water or substrate limitation for plants and soil microbes. Interestingly, there were positive correlations between responses of MBC and APB to IPPT (Figure 4C). We speculated that increased precipitation treatments probably increased litter inputs and decomposition rates because of the profuse leaf growth and speedy turnover so that soil microbes might be provided with recent photosynthates (Austin and Vitousek, 2000; Yahdjian et al., 2006). Of course, we cannot rule out the possibility that moderate increase in soil moisture may stimulate exudation of root metabolites that can, as enzymes, cause speedy degradation of organic matters and release of labile carbon (Dijkstra and Cheng, 2007; Canarini et al., 2019). Our results highlighted that the relationships between microbial biomass and plant production could not be simply linear when precipitation regimes change. The association between roots and soil microbes could be much stronger under increased precipitation conditions, while under decreased precipitation, stresses for microbial biomass were likely to be buffered because of drought-induced rhizodeposition.

## Conclusions

This meta-analysis produced several key findings and filled knowledge gaps for combined responses of plant-soil systems to precipitation changes. Grassland responses to altered precipitation varied in the magnitude between different compartments, with greater APB responses than BPB or MBC responses. DPPT increased biomass allocation to roots for acquiring water, while IPPT increased biomass allocation to shoots for light capture. We detected a trade-off between response ratios of APB and BPB to DPPT, supporting the optimal allocation theory. Shifts in root biomass allocation probably neutralized the effects of precipitation changes on roots. Under DPPT, increased root allocation probably buffered drought stress for soil microbes and led to neutral responses of microbial biomass C. Our study provides evidence that plant biomass allocation mediates asynchrony between APB, BPB, and MBC, and emphasizes that forecasting the consequences of precipitation changes for grassland systems requires testing the effects on the plant-soil system as a whole.

## Data Availability Statement

The original contributions presented in the study are included in the article/Supplementary Material, further inquiries can be directed to the corresponding author/s.

## Author Contributions

NX and CZ designed the study, CZ and NX performed data collection and conducted statistical analyses, and NX and CZ wrote the manuscript. Both authors contributed to the article and approved the submitted version.

## Conflict of Interest

The authors declare that the research was conducted in the absence of any commercial or financial relationships that could be construed as a potential conflict of interest.

## Abbreviations

APB: aboveground plant biomass
BPB: belowground plant biomass
MBC: microbial biomass carbon
DPPT: decreased precipitation
IPPT: increased precipitation.
