# Supplementary material for: Precipitation Changes Regulate Plant and Soil Microbial Biomass Via Plasticity in Plant Biomass Allocation in Grasslands: A Meta-Analysis
Source: Front Plant Sci. 2021 Feb 25;12:614968. doi: 10.3389/fpls.2021.614968 (PMC7947227; doi:10.3389/fpls.2021.614968)
Supplement: Supplementary file 1 [file Data_Sheet_1.docx]

**Appendix S1 Reference for NPP**

| 1. Brueck, H., Erdle, K., Gao, Y. *et al.* (2010). Effects of N and water supply on water use-efficiency of a semiarid grassland in Inner Mongolia. *Plant Soil* **328,** 495–505. |
| --- |
| 1. Byrne, K. M., Lauenroth, W. K., & Adler, P. B. (2013). Contrasting effects of precipitation manipulations on production in two sites within the central grassland region, usa. *Ecosystems,* *6*(6), 1039-1051. |
| 1. Canarini, A., Mariotte, P., Ingram, L., Merchant, A., & Dijkstra, F. A. (2017). Mineral-associated soil carbon is resistant to drought but sensitive to legumes and microbial biomass in an australian grassland. *Ecosystems*. 21, 349–335. |
| 1. Carlyle, C. N., Fraser, L. H., & Turkington, R. (2014). Response of grassland biomass production to simulated climate change and clipping along an elevation gradient. *Oecologia,* *174*(3), 1065-1073. |
| 1. Chelli, S., Canullo, R., Campetella, G., Schmitt, A. O., Bartha, Sándor, & Cervellini, M., et al. (2016). The response of sub-mediterranean grasslands to rainfall variation is influenced by early season precipitation. *Applied Vegetation Science*. 19, 611–619. |
| 1. Chen, Q., Hooper, D. U., Li, H., Gong, X. Y., Peng, F., Wang, H., ... & Lin, S. (2017). Effects of resource addition on recovery of production and plant functional composition in degraded semiarid grasslands. *Oecologia*, *184*(1), 13-24. |
| 1. Chimner, R. A., Welker, J. M., Morgan, J., LeCain, D., Reeder, J. (2010) Experimental manipulations of winter snow and summer rain influence ecosystem carbon cycling in a mixed‐grass prairie, Wyoming, USA. *Ecohydrology*. 3, 284-293 |
| 1. Chou, W. W., Silver, W. L., Jackson, R. D., Thompson, A. W., & Allen-Diaz, B. (2008). The sensitivity of annual grassland carbon cycling to the quantity and timing of rainfall. *Global Change Biology,* *14*(6), 1382-1394. |
| 1. Cole, A. J., Griffiths, R. I., Ward, S. E., Whitaker, J., Ostle, N. J., & Bardgett, R. D. (2019). Grassland biodiversity restoration increases resistance of carbon fluxes to drought. *Journal of Applied Ecology*. 56, 1806–1816. |
| 1. Deng, Q., Aras, S., Yu, C. L., Dzantor, E. K., Fay, P. A., Luo, Y., ... & Hui, D. (2017). Effects of precipitation changes on aboveground net primary production and soil respiration in a switchgrass field. *Agriculture, Ecosystems & Environment*, *248*, 29-37. |
| 1. Denton, E. M., Dietrich, J. D., Smith, M. D., & Knapp, A. K. (2016). Drought timing differentially affects above- and belowground productivity in a mesic grassland. P*lant Ecology,* *218*(3), 1-12. |
| 1. Dukes, J. S., Chiariello, N. R., Cleland, E. E., Moore, L. A., Shaw, M. R., & Thayer, S., et al. (2005). Responses of grassland production to single and multiple global environmental changes. *PLoS Biology,* *3*(10), e319. |
| 1. Evans, S. E., & Burke, I. C. (2013). Carbon and nitrogen decoupling under an 11-year drought in the shortgrass steppe. *Ecosystems,* *16*(1), 20-33. |
| 1. Eze, S., Palmer, S. M., & Chapman, P. J. (2018). Negative effects of climate change on upland grassland productivity and carbon fluxes are not attenuated by nitrogen status. *Science of The Total Environment,* *637-638*, 398-407. |
| 1. Fay, P. A., Carlisle, J. D., Knapp, A. K., Blair, J. M., & Collins, S. L. (2003). Productivity responses to altered rainfall patterns in a c4-dominated grassland. *Oecologia,* *137*(2), 245-251. |
| 1. Fiala, K., Tůma, I., Holub, P. (2012). Interannual Variation in Root Production in Grasslands Affected by Artiﬁcially Modiﬁed Amount of Rainfall. The Scientiﬁc World Journal. doi:10.1100/2012/805298 |
| 1. Gao, Y.Z., Chen, Q., Lin, S. et al. (2011). Resource manipulation effects on net primary production, biomass allocation and rain-use efficiency of two semiarid grassland sites in Inner Mongolia, China. Oecologia 165, 855–864. |
| 1. Gilgen, A. K., Buchmann, N. (2009). Response of temperate grasslands at different altitudes to simulated summer drought differed but scaled with annual precipitation. *Biogeosciences Discuss.*, 6, 5217–5250. |
| 1. Gong, J., Xu, S., Wang, Y. *et al.* Effect of irrigation on the soil respiration of constructed grasslands in Inner Mongolia, China. *Plant Soil* **395,** 159–172 (2015). |
| 1. Skinner, R.H., Hanson, J. D., & Hutchinson, G. L. (2002). Response of c3and c4grasses to supplemental summer precipitation. *Journal of Range Management,* *55*(5), 517-522. |
| 1. Harpole, W. S., Potts, D. L., & Suding, K. N. (2007). Ecosystem responses to water and nitrogen amendment in a california grassland. *Global Change Biology,* *13*(11), 2341-2348. |
| 1. Henry, H. A. L., Abedi, M., Alados, Concepción L., Beard, K. H., Fraser, L. H., & Jentsch, A., et al. (2018). Increased soil frost versus summer drought as drivers of plant biomass responses to reduced precipitation: results from a globally coordinated field experiment. *Ecosystems*. 21, 1432–1444 |
| 1. Henry, H. A. L., Chiariello, N. R. , Vitousek, P. M. , Mooney, H. A. , & Field, C. B. . (2006). Interactive effects of fire, elevated carbon dioxide, nitrogen deposition, and precipitation on a california annual grassland. *Ecosystems,* *9*(7), 1066-1075. |
| 1. Hoeppner, S. S., Dukes, J. S. (2012). Interactive responses of old-ﬁeld plant growth and composition to warming and precipitation. *Global Change Biology*, 18, 1754–1768 |
| 1. Holub, P., Fabšičová, M., Tůma, I., Záhora, J., Fiala, K. (2012). Effects of artificially varying amounts of rainfall on two semi‐natural grassland types. *Journal of Vegetation Science*, 24, 518-529. |
| 1. Hoover, D. L. , Knapp, A. K. , & Smith, M. D. . (2014). Resistance and resilience of a grassland ecosystem to climate extremes. *Ecology,* *95*(9), 2646–2656. |
| 1. Hoover, D. L. , Knapp, A. K. , & Smith, M. D. . (2016). The immediate and prolonged effects of climate extremes on soil respiration in a mesic grassland. *Journal of Geophysical Research Biogeosciences.* 121, 1034–1044, |
| 1. Knapp, A. K. , Briggs, J. M. , & Koelliker, J. K. . (2001). Frequency and extent of water limitation to primary production in a mesic temperate grassland. *Ecosystems,* *4*(1), 19-28. |
| 1. Koerner, S. E. , & Collins, S. L. . (2014). Interactive effects of grazing, drought, and fire on grassland plant communities in north america and south africa. *Ecology,* *95*(1), 98-109. |
| 1. Lawrence, B., Flanagan, and, Eric, & J., et al. (2013). Response of plant biomass and soil respiration to experimental warming and precipitation manipulation in a northern great plains grassland. Agricultural and Forest Meteorolog*y,* 173, 40–52 |
| 1. Li, H., Yang, S., Xu, Z., Yan, Q., Li, X., et al. (2017). Responses of soil microbial functional genes to global changes are indirectly influenced by aboveground plant biomass variation. Soil Biology and Biochemistry. 104, 18-29. 2. Li, H., Xu, Z., Yang, S., Li, X., Top, E. M., Wang, R., ... & Jiang, Y. (2016). Responses of Soil Bacterial Communities to Nitrogen Deposition and Precipitation Increment Are Closely Linked with Aboveground Community Variation. Microbial Ecology, 71(4), 974-989. |
| 1. Li, J., Huang, Y. , Xu, F. , Wu, L. , Chen, D. , & Bai, Y. . (2018). Responses of growing-season soil respiration to water and nitrogen addition as affected by grazing intensity. *Functional Ecology*. 32, 1890–1901. |
| 1. Li, K., Liu, X., Song, L. *et al.* (2015) Response of alpine grassland to elevated nitrogen deposition and water supply in China. *Oecologia* **177,** 65–72. |
| 1. Lin, L., Zhu, B. , Chen, C. , Zhang, Z. , Wang, Q. B. , & He, J. S. . (2016). Precipitation overrides warming in mediating soil nitrogen pools in an alpine grassland ecosystem on the Tibetan plateau. *Scientific Reports,* *6*, 31438. |
| 1. Liu, H. , Mi, Z. , Lin, L. , Wang, Y. , Zhang, Z. , & Zhang, F. , et al. (2018). Shifting plant species composition in response to climate change stabilizes grassland primary production. *Proceedings of the National Academy of Sciences,* *115*(16), 4051-4056. |
| 1. Lü Xiao-Tao, Kong, D. L. , Pan, Q. M. , & Han, S. X. G. . (2012). Nitrogen and water availability interact to affect leaf stoichiometry in a semi-arid grassland. *Oecologia,* *168*(2), 301-310. |
| 1. Ma, L. , Huang, W. , Guo, C. , Wang, R. , & Xiao, C. . (2012). Soil microbial properties and plant growth responses to carbon and water addition in a temperate steppe: the importance of nutrient availability. *Plos One,* 7(4), e35165. |
| 1. Miranda, J. D., Armas, C., Padilla, F. M., & Pugnaire, F. I. (2011). Climatic change and rainfall patterns: Effects on semi-arid plant communities of the Iberian Southeast. *Journal of Arid Environments*, 75(12), 1302-1309. |
| 1. Prechsl, U.E., Burri, S., Gilgen, A.K. *et al.* (2015) No shift to a deeper water uptake depth in response to summer drought of two lowland and sub-alpine C3-grasslands in Switzerland. *Oecologia* **177,** 97–111. |
| 1. Prevéy, Janet S., Seastedt, T. R. , & Wilson, S. . (2014). Seasonality of precipitation interacts with exotic species to alter composition and phenology of a semi-arid grassland. *Journal of Ecology,* *102*(6), 1549-1561. 2. Shao, P., He, H., Zhang, X., Xie, H., Bao, X., & Liang, C. (2018). Responses of microbial residues to simulated climate change in a semiarid grassland. *Science of The Total Environment,* *644*, 1286-1291. |
| 1. Shen, Y., Chen, W., Yang, G., Yang, X., Liu, N., & Sun, X., et al. (2016). Can litter addition mediate plant productivity responses to increased precipitation and nitrogen deposition in a typical steppe? *Ecological research,* *31*(4), 579-587. |
| 1. Sherry, R. A., Weng, E., Arnone Iii, J. A., Johnson, D. W., Schimel, D. S., & Verburg, P. S., et al. (2008). Lagged effects of experimental warming and doubled precipitation on annual and seasonal aboveground biomass production in a tallgrass prairie. *Global Change Biology,* *14*(12), 2923-2936. |
| 1. Shi, F., Chen, H., Chen, H., Wu, Y., & Wu, N. (2012). The combined effects of warming and drying suppress CO2 and N2O emission rates in an alpine meadow of the eastern Tibetan Plateau. *Ecological Research*, 27(4), 725-733. |
| 1. Song, W., Chen, S., Wu, B., Zhu, Y., Zhou, Y., & Li, Y., et al. (2012). Vegetation cover and rain timing co-regulate the responses of soil CO_2_ efflux to rain increase in an arid desert ecosystem. *Soil Biology & Biochemistry,* *49*, 114-123. |
| 1. Spence, L.A., Liancourt, P., Boldgiv, B., Petraitis, P.S., Casper, B.B. (2016). Short‐term manipulation of precipitation in Mongolian steppe shows vegetation influenced more by timing than amount of rainfall. Journal of Vegetation Science. 27, 249–258. |
| 1. Su, F., Wei, Y., Wang, F., Guo, J., Zhang, J., & Wang, Y., et al. (2019). Sensitivity of plant species to warming and altered precipitation dominates the community productivity in a semiarid grassland on the loess plateau. *Ecology and Evolution*, 7628-7638. |
| 1. SUTTLE, K. B., Thomsen, M. A. (2007). Climate change and grassland restoration in California: lessons from six years of rainfall manipulation in a north coast grassland. Madroño 54, 225-233. |
| 1. Thomey, M. L., Collins, S. L., Vargas, R., Johnson, J. E., Brown, R. F. , & Natvig, D. O. , et al. (2011). Effect of precipitation variability on net primary production and soil respiration in a chihuahuan desert grassland. *Global Change Biology,* *17*(4), 1505-1515. |
| 1. Wilcox, K. R. , Blair, J. M. , & Knapp, A. K. . (2016). Stability of grassland soil c and n pools despite 25?years of an extreme climatic and disturbance regime. *Journal of Geophysical Research Biogeosciences,* *121*(7), 1934-1945. |
| 1. Wilcox, K. R. , Fischer, J. C. V. , Muscha, J. M. , Petersen, M. K. , & Knapp, A. K. . (2014). Contrasting above- and belowground sensitivity of three great plains grasslands to altered rainfall regimes. *Global Change Biology,* *21*(1), 335-344. |
| 1. Xiao, C., Janssens, I. A., Liu, P., Zhou, Z., Sun, O.J. (2007). Irrigation and enhanced soil carbon input effects on below-ground carbon cycling in semiarid temperate grasslands. *New Phytologist,* *174*(4), 835-846. |
| 1. Xu, X., Sherry, R. A., Niu, S., Li, D., & Luo, Y. (2013). Net primary productivity and rain-use efficiency as affected by warming, altered precipitation, and clipping in a mixed-grass prairie. *Global Change Biology,* *19*(9), 2753-2764. |
| 1. Xu, X., Shi, Z., Chen, X., Lin, Y., Niu, S., & Jiang, L., et al. (2016). Unchanged carbon balance driven by equivalent responses of production and respiration to climate change in a mixed‐grass prairie. *Global Change Biology,* *22*(5), 1857-1866. |
| 1. Xu, Z., Hou, Y. , Zhang, L. , Liu, T. , & Zhou, G. . (2016). Ecosystem responses to warming and watering in typical and desert steppes. *Scientific Reports,* *6*, 34801. |
| 1. Xu, Z., Ren, H. , Li, M. H. , Brunner, I. , Yin, J. , & Liu, H. , et al. (2016). Experimentally increased water and nitrogen affect root production and vertical allocation of an old-field grassland. *plant & soil,* *412*(1-2), 1-12. |
| 1. Yahdjian, L. , & Sala, O. E. . (2006). Vegetation structure constrains primary production response to water availability in the patagonian steppe. *Ecology,* *87*(4), 952-962. |
| 1. Yan, L., Chen, S., Huang, J., & Lin, G. (2011). Water regulated effects of photosynthetic substrate supply on soil respiration in a semiarid steppe. *Global Change Biology*, 17(5), 1990-2001. |
| 1. Zhang, F., Quan, Q. , Song, B. , Sun, J. , Chen, Y. , & Zhou, Q. , et al. (2017). Net primary productivity and its partitioning in response to precipitation gradient in an alpine meadow. *Scientific Reports,* *7*(1), 15193. |
| 1. Zhang, H., Liu, H., Zhao, J., Wang, L., Li, G., et al. (2017). Elevated precipitation modifies the relationship between plant diversity and soil bacterial diversity under nitrogen deposition in Stipa baicalensis steppe. Applied Soil Ecology. 119, 345–353 |
| 1. Zhang, H., Yu, H., et al. (2019). Aboveground net primary productivity not co2 exchange remain stable under three timing of extreme drought in a semi-arid steppe. *PloS one,* 14(3), e0214418. |
| 1. Zhang, L., Xie, Z. , Zhao, R. , & Zhang, Y. (2018). Plant, microbial community and soil property responses to an experimental precipitation gradient in a desert grassland. *Applied Soil Ecology,* 127, 87-95. |
| 1. Zhang, X., Tan, Y. , Zhang, B. , Li, A. , Daryanto, S. , & Wang, L. , et al. (2017). The impacts of precipitation increase and nitrogen addition on soil respiration in a semiarid temperate steppe. *Ecosphere,* *8*(1), e01655. 2. Zhang, X., Zhai, P., Huang, J., Zhao, X., Dong, K. (2018). Responses of ecosystem water use efficiency to spring snow and summer water addition with or without nitrogen addition in a temperate steppe. PLoS ONE 13(3): e0194198 |

**Appendix S2 Reference for MBC**

| 1. Bell, C. W., Tissue, D. T., Loik, M. E., Wallenstein, M. D., & Zak, J. C. (2013). Soil microbial and nutrient responses to 7 years of seasonally altered precipitation in a chihuahuan desert grassland. *Global Change Biology,* *20*(5), 1657-1673. |
| --- |
| 1. Canarini, A., Mariotte, P., Ingram, L., Merchant, A., & Dijkstra, F. A. (2017). Mineral-associated soil carbon is resistant to drought but sensitive to legumes and microbial biomass in an australian grassland. *Ecosystems*. DOI: 10.1007/s10021-017-0152-x |
| 1. Cole, A. J., Griffiths, R. I., Ward, S. E., Whitaker, J., Ostle, N. J., & Bardgett, R. D. (2019). Grassland biodiversity restoration increases resistance of carbon fluxes to drought. *Journal of Applied Ecology,* 56, 1806–1816. |
| 1. Eisenhauer, N., Cesarz, S., Koller, R., Worm, K., & Reich, P. B. (2012). Global change belowground: impacts of elevated CO2, nitrogen, and summer drought on soil food webs and biodiversity. *Global Change Biology*, 18(2), 435-447. |
| 1. Eze, S., Palmer, S. M., & Chapman, P. J. (2018). Negative effects of climate change on upland grassland productivity and carbon fluxes are not attenuated by nitrogen status. *Science of The Total Environment,* *637-638*, 398-407. |
| 1. Gong, J. R., Xu, S., Wang, Y., Luo, Q., Liu, M., & Zhang, W. (2015). Effect of irrigation on the soil respiration of constructed grasslands in Inner Mongolia, China. *Plant and Soil*, 159-172. |
| 1. Hartmann, A. A., & Niklaus, P. A.. (2012). Effects of simulated drought and nitrogen fertilizer on plant productivity and nitrous oxide (n2o) emissions of two pastures. *Plant & Soil,* *361*(1-2), 411-426. |
| 1. Huang, G., Li, Y. , & Su, Y. G. . (2015). Divergent responses of soil microbial communities to water and nitrogen addition in a temperate desert. *Geoderma, 251*-*252*, 55-64. |
| 1. Huang, G. , Li, Y. , & Su, Y. G. . (2018). Differential seasonal effects of water addition and nitrogen fertilization on microbial biomass and diversity in a temperate desert. *Catena, 161*, 27–36. |
| 1. Li, H., Xu, Z., Yang, S., Li, X., Top, E. M., Wang, R., ... & Jiang, Y. (2016). Responses of Soil Bacterial Communities to Nitrogen Deposition and Precipitation Increment Are Closely Linked with Aboveground Community Variation. *Microbial Ecology*, 71(4), 974-989. |
| 1. Li, H., Yang, S., Xu, Z., Yan, Q., Li, X., et al. (2017). Responses of soil microbial functional genes to global changes are indirectly influenced by aboveground plant biomass variation. *Soil Biology and Biochemistry, 104*, 18-29. |
| 1. Liu, W. , Zhang, Z. , & Wan, S. (2009). Predominant role of water in regulating soil and microbial respiration and their responses to climate change in a semiarid grassland. *Global Change Biology,* *15*(1), 184-195. |
| 1. Ma, L. , Huang, W. , Guo, C. , Wang, R. , & Xiao, C. (2012). Soil microbial properties and plant growth responses to carbon and water addition in a temperate steppe: the importance of nutrient availability. *Plos One,* 7(4), e35165. |
| 1. Reinsch, S. , Michelsen, A. , Sárossy, Zsuzsa, Egsgaard, H. , Schmidt, I. K. , & Jakobsen, I. , et al. (2014). Short-term utilization of carbon by the soil microbial community under future climatic conditions in a temperate heathland. *Soil Biology and Biochemistry,* *68*, 9-19. |
| 1. Shao, P., He, H., Zhang, X., Xie, H., Bao, X., & Liang, C. (2018). Responses of microbial residues to simulated climate change in a semiarid grassland. *Science of The Total Environment,* *644*, 1286-1291. |
| 1. Shi, F., Chen, H., Chen, H., Wu, Y., & Wu, N. (2012). The combined effects of warming and drying suppress CO2 and N2O emission rates in an alpine meadow of the eastern Tibetan Plateau. *Ecological Research*, 27(4), 725-733. |
| 1. Song, W. , Chen, S. , Wu, B. , Zhu, Y. , Zhou, Y. , & Li, Y. , et al. (2012). Vegetation cover and rain timing co-regulate the responses of soil co2 efflux to rain increase in an arid desert ecosystem. *soil biology & biochemistry,* *49*, 114-123. |
| 1. Tian, J. , Wei, K. , Condron, L. M. , Chen, Z. , Xu, Z. , & Feng, J. , et al. (2017). Effects of elevated nitrogen and precipitation on soil organic nitrogen fractions and nitrogen-mineralizing enzymes in semi-arid steppe and abandoned cropland. *Plant & Soil*. 417, 217–229. |
| 1. Vogel, A. , Eisenhauer, N. , Weigelt, A. , & Scherer-Lorenzen, M. (2013). Plant diversity does not buffer drought effects on early-stage litter mass loss rates and microbial properties. *Global Change Biology,* *19*(9), 2795-2803. |
| 1. Wang, R., Filley, T. R., Xu, Z., Wang, X., Li, M., Zhang, Y., ... & Jiang, Y. (2014). Coupled response of soil carbon and nitrogen pools and enzyme activities to nitrogen and water addition in a semi-arid grassland of Inner Mongolia. *Plant and Soil*, 381(1), 323-336. |
| 1. Wang, Z. , Silva, L. C. R. , Sun, G. , Luo, P. , Mou, C. , & Horwath, W. R. (2015). Quantifying the impact of drought on soil-plant interactions: a seasonal analysis of biotic and abiotic controls of carbon and nutrient dynamics in high-altitudinal grasslands. *Plant and Soil,* *389*(1-2), 59-71. |
| 1. Xiao, Chunwang, Janssens, Ivan A., Liu, Ping, Zhou, Zhiyong, Sun, Osbert J. (2007). Irrigation and enhanced soil carbon input effects on below-ground carbon cycling in semiarid temperate grasslands. *New Phytologist,* *174*(4), 835-846. |
| 1. Xu, Z. , Hou, Y. , Zhang, L. , Liu, T. , & Zhou, G. (2016). Ecosystem responses to warming and watering in typical and desert steppes. *Scientific Reports,* *6*, 34801. |
| 1. Yan, L. , Chen, S. , Huang, J. , & Lin, G. (2010). Differential responses of auto- and heterotrophic soil respiration to water and nitrogen addition in a semiarid temperate steppe. *Global Change Biology,* *16*(8), 2345-2357. |
| 1. Zhang, B. , Li, S. , Chen, S. , Ren, T. , & Han, X. (2016). Arbuscular mycorrhizal fungi regulate soil respiration and its response to precipitation change in a semiarid steppe. *Scientific Reports,* *6*, 19990. |
| 1. Zhang, L. , Xie, Z. , Zhao, R. , & Zhang, Y. (2018). Plant, microbial community and soil property responses to an experimental precipitation gradient in a desert grassland. *Applied Soil Ecology*. 127, 87-95 |
| 1. Zhang, N., Liu, W., Yang, H., Yu, X., & Jessica L. M., et al. (2013). Soil microbial responses to warming and increased precipitation and their implications for ecosystem c cycling. *Oecologia,* *173*(3), 1125-1142. |
| 1. Zhang, X., Tan, Y., Zhang, B., Li, A., Daryanto, S., & Wang, L., et al. (2017). The impacts of precipitation increase and nitrogen addition on soil respiration in a semiarid temperate steppe. *Ecosphere,* *8*(1), e01655. |
| 1. Zhao, C., Miao, Y., Yu, C., Zhu, L., Wang, F., & Jiang, L., et al. (2016). Soil microbial community composition and respiration along an experimental precipitation gradient in a semiarid steppe. *Scientific Reports,* *6*, 24317. |
| 1. Zhou, X., Chen, C., Wang, Y., Xu, Z., Han, H., & Li, L., et al. (2013). Warming and increased precipitation have differential effects on soil extracellular enzyme activities in a temperate grassland. *Science of the Total Environment,* *444*, 552-558. |

**Figure S1**

**
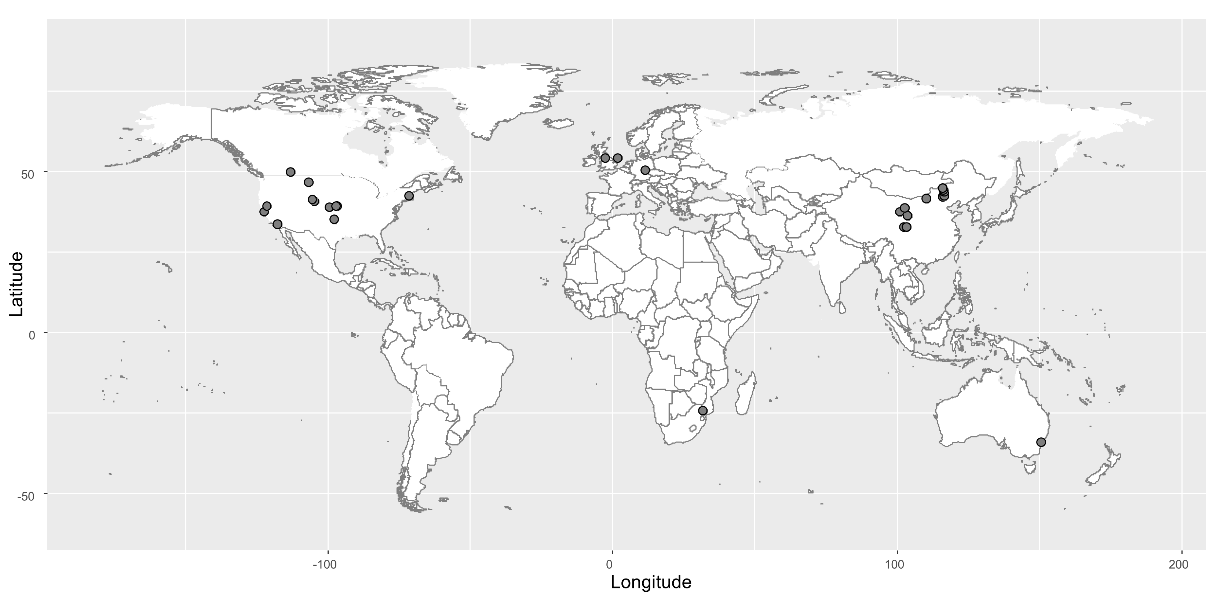
**

**Figure S1** Geographical locations for the 112 case studies included in in the meta-analysis. Locations as gray dots. Gray dots may represent multiple case studies.

**Figure S2**


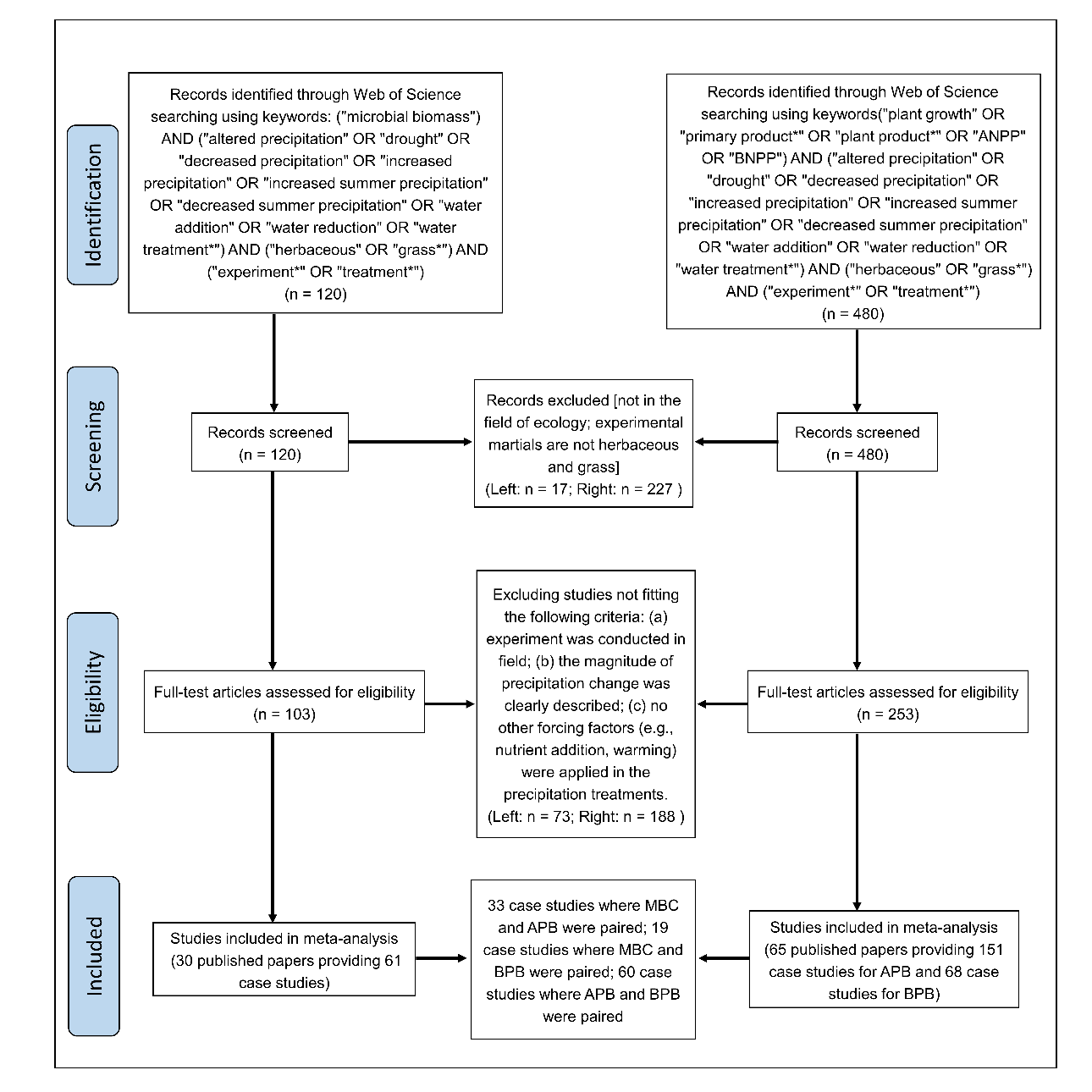


**Figure S2** PRISMA ﬂow diagram showing the procedure used for literature searching and selecting


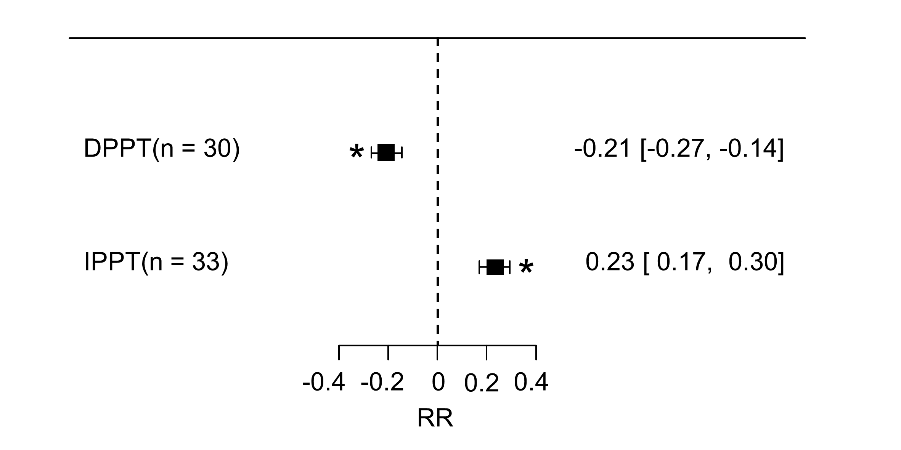


**Fig. S3** Response ratio of soil moisture in response to DPPT and IPPT. The error bars represent 95% confidence intervals. Asterisks indicate average response ratios as they differ from zero (*P* < 0.05). The dotted line indicates the effect size of zero. DPPT = decreased precipitation; IPPT = increased precipitation.


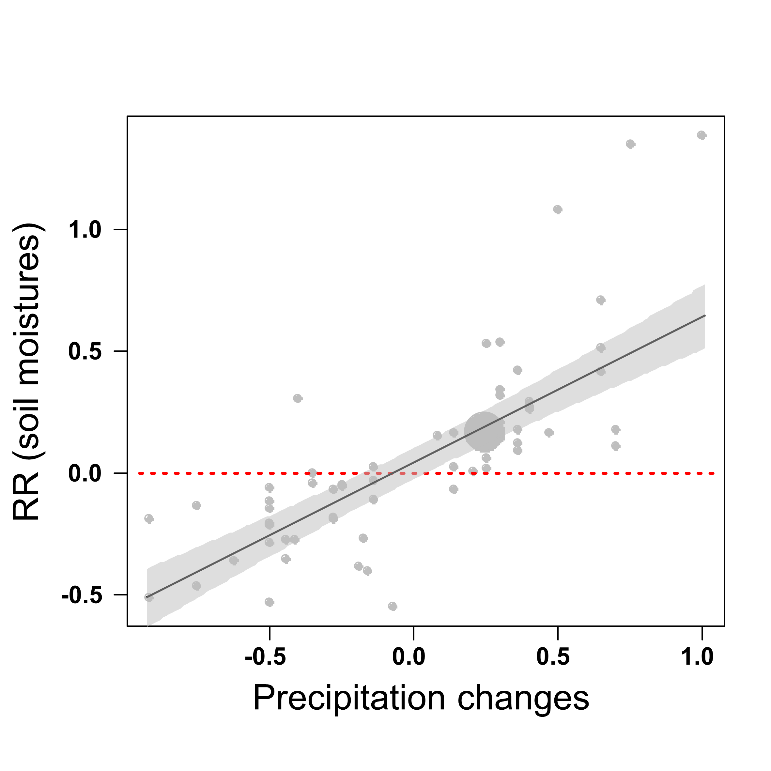


**Fig. S4** Relationships between precipitation changes and effect size of soil moistures to altered precipitation. The shaded areas represent 95% confident intervals.

**Table S1** Publication bias tests for the microbial variables based on the rank correlation test for the funnel plot asymmetry. Publication bias was detected if the *P*-value was lower than 0.05.

| Variable | Precipitation decrease | | | Precipitation increase | | |
| --- | --- | --- | --- | --- | --- | --- |
|  | *N* | Kendall's tau | *P* | *N* | Kendall's tau | *P* |
| MBC | 12 | -0.3333 | 0.1526 | 35 | 0.0387 | 0.7565 |
| APB | 42 | -0.1766 | 0.0995 | 74 | 0.0490 | 0.5378 |
| BPB | 35 | -0.0101 | 0.9321 | 54 | -0.0687 | 0.4646 |
| D(APB/BPB) | 34 | -0.2319 | 0.0539 | 54 | -0.0771 | 0.4117 |

**Table S2** Relationships among RR of MBC, APB and BPB

|  |  | *N* | Slope | *P* | Intercept |
| --- | --- | --- | --- | --- | --- |
| DPPT |  |  |  |  |  |
|  | APB vs BPB | 34 | -0.38142 | 0.0384 | -0.05154 |
|  | APB vs MBC | 11 | -0.3257 | 0.2983 | -0.3836 |
|  | BPB vs MBC | 4 | - | - | - |
| IPPT |  |  |  |  |  |
|  | APB vs BPB | 54 | 0.28782 | 0.08868 | -0.01026 |
|  | APB vs MBC | 35 | 0.5754 | 0.002331 | 0.1311 |
|  | BPB vs MBC | 15 | -0.2281 | 0.29630 | 0.3419 |
